# Properties of cationic monosubstituted tetraalkylammonium cyclodextrin derivatives – their stability, complexation ability in solution or when deposited on solid anionic surface

**DOI:** 10.3762/bjoc.11.20

**Published:** 2015-02-02

**Authors:** Martin Popr, Sergey K Filippov, Nikolai Matushkin, Juraj Dian, Jindřich Jindřich

**Affiliations:** 1 Department of Organic Chemistry, Faculty of Science, Charles University in Prague, Hlavova 8, 128 40, Prague 2, Czech Republic; 2 Institute of Macromolecular Chemistry AS CR, v. v. i., Heyrovskeho nam. 22, 16206, Prague 6, Czech Republic; 3 Department of Chemical Physics and Optics, Faculty of Mathematics and Physics, Charles University in Prague, Ke Karlovu 3, 121 16, Prague 2, Czech Republic

**Keywords:** cyclodextrins, inclusion properties, solid surface, tetraalkylammonium derivatives, thermal stability

## Abstract

The thermal stability of the monosubstituted cationic cyclodextrin (CD) derivatives PEMEDA-β-CD and PEMPDA-β-CD, which differ in their substituent linker length (ethylene and propylene, respectively), was studied via ^1^H NMR experiments. PEMPDA-β-CD exhibited higher resistance towards the Hofmann degradation and was chosen as a more suitable host molecule for further studies. Inclusion properties of PEMPDA-β-CD in solution with a series of simple aromatic guests (salicylic acid, *p*-methoxyphenol and *p*-nitroaniline) were determined by isothermal titration calorimetry (ITC) and compared to the native β-CD. Permanently charged cationic CD derivatives were successfully deposited on the anionic solid surface of polymeric Nafion^®^ 117 membrane via electrostatic interactions. Deposition kinetics and coverage of the surface were determined by ELSD. Finally, the ability of the CD derivatives bound to the solid surface to encapsulate aromatic compounds from aqueous solution was measured by UV–vis spectroscopy. The obtained results are promising for future industrial applications of the monosubstituted β-CD derivatives, because the preparation of cationic CD derivatives is applicable in large scale, without the need of chromatographic purification. Their ionic deposition on a solid surface is simple, yet robust and a straightforward process as well.

## Introduction

Cyclodextrins (CDs) are a very interesting group of natural macrocyclic carbohydrates, which were first described by Villiers in 1891 [1]. They are composed of α-(1→4)-linked D-glucopyranose units forming a cycle with the shape of a hollow truncated cone. Naturally occurring CDs are α-, β- and γ-CD with 6, 7 and 8 glucopyranose units, respectively. Their inner cavity, which is relatively hydrophobic, creates a proper environment for encapsulation of a wide range of organic and inorganic molecules [2]. Therefore CDs and their derivatives are most often utilized in pharmaceutical industry for drug solubilization [3] and delivery [4].

Recently, we have published an article on the synthesis of a complete series of cationic monosubstituted tetraalkylammonium CD derivatives with one, two or three permanent positive charges [5]. The described preparation is straightforward and relatively high-yielding in contrast to other conventional methods which usually suffer from low yields and the need of chromatographic purification of reaction mixtures in order to obtain pure isomers [6]. This makes our approach expandable to large scale and potentially suitable for industrial applications. Previously, it has been described, that cationic CD derivatives with permanent positive charge can be synthesized [7] and used as chiral selectors in capillary zone electrophoresis [8–12] and also as catalysts of chemical reactions [13–15].

The main goal of our research is to explore the possibility of binding of the monosubstituted cationic CD derivatives to an anionic surface by simple electrostatic interactions. Such an assembly could be potentially used for transdermal transportation of encapsulated active compounds across the epidermis with the possibility of controlling the rate of the transport by modulating the CD carrier, which is bound on the surface. The obtained results can be possibly used for designing a new topical drug formulation such as “smart” plasters or bandages capable of prolonged release of the antiseptic drug. There are several examples of deposition of CD derivatives onto a solid support in the literature. The most common immobilization method is binding of various alkylthio-CDs onto a gold surface [16–18]. This approach offers self-assembled monolayers (SAMs) which can be utilized for derivatization of gold electrodes for the construction of electrochemical sensors [19–20] or even for the development of molecular printboards [21]. These results imply that the structure of CD derivatives strongly influences the properties of the SAMs [16]. Similar assemblies/films can be prepared on glass surface, by depositing different CD-siloxanes [22–23].

Recently, some examples of anchoring CDs onto the solid support via ionic interactions were reported. Most of them describe deposition of cationic CD polymers, where the CD units are randomly crosslinked by suitable reagents. Systems for separation of gases, composed of cationic CD pyridinium polymer and a Nafion^®^ membrane were reported by Grossi and coworkers [24]. The Kusumocahyo group studied separation abilities of cationic CD copolymers deposited on an anionic Nafion^®^ membrane, which showed good selectivity toward butanol isomers [25]. Multilayered assemblies of anionic sulfonated CD derivatives and cationic polyelectrolytes were also prepared by a layer-by-layer strategy and used for the construction of an optical sensor for litocholic acid [26]. Similar approaches of ionic self-assembly with CDs were used for the preparation of nanoparticles with external CD trigger [27], new material nanostructures [28], polyelectrolyte-surfactant complexes which yield new types of solid mesomorphous materials [29], or membranes with size-selective transport of aromatic compounds [30].

In this paper we report on a comprehensive study of the properties of the novel monosubstituted cationic CD derivative PEMPDA-β-CD (PErMethylated PropyleneDiAmine substituted). Its thermal stability is compared to the previously reported analogue PEMEDA-β-CD (PErMethylated EthyleneDiAmine substituted). Its binding ability toward selected aromatic guests is compared to the native β-CD at different pH. Deposition of PEMPDA-β-CD on the surface of an anionic Nafion^®^ 117 membrane via ionic self-assembly was also studied along with the determination of sorption kinetics and surface coverage. Finally, the ability of the PEMPDA-β-CD bound to the solid surface to accommodate aromatic guests from aqueous solution into the cavities was explored.

## Results and Discussion

### Thermal stability of PEMEDA- and PEMPDA-β-CD

**Scheme 1:**
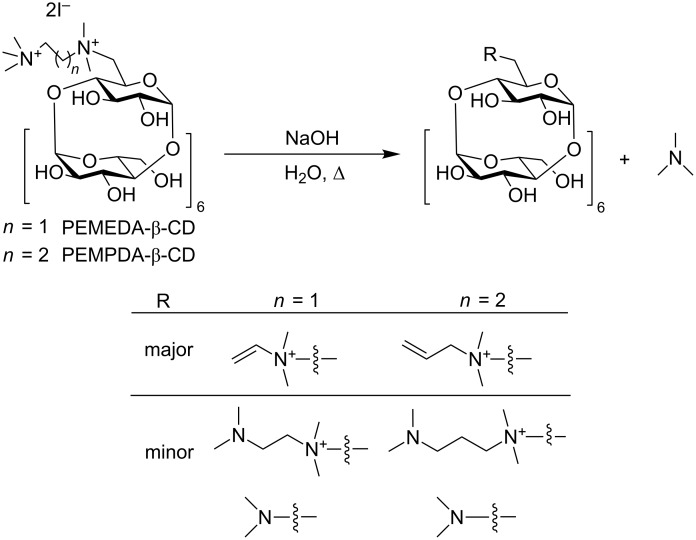
Thermal decomposition of PEMEDA- and PEMPDA-β-CD with the decomposition products as characterized by MS.

**Figure 1:**
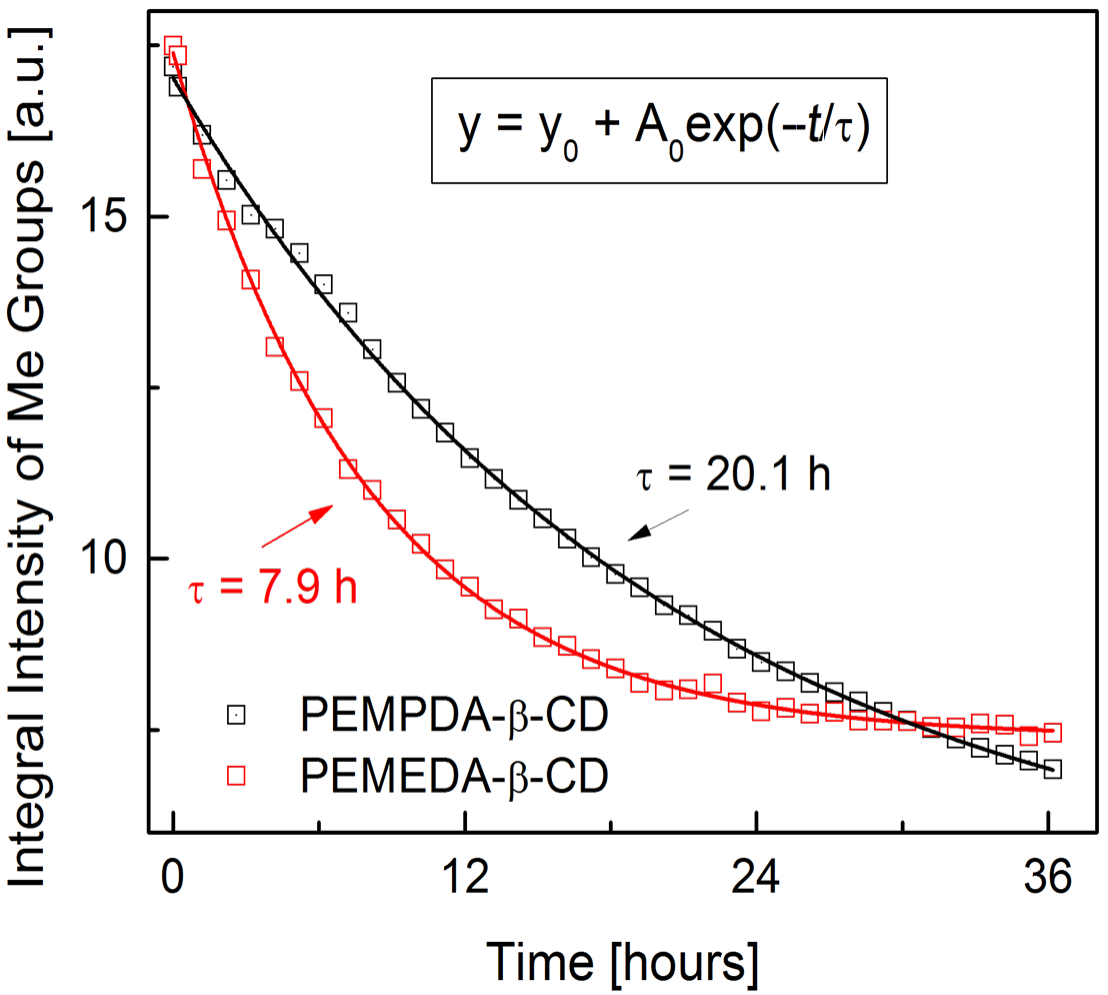
Decomposition kinetics of PEMEDA- and PEMPDA-β-CD at 50 °C as determined by ^1^H NMR thermal experiments.

It is generally known, that quaternary ammonium salts exhibit a low stability in basic environment at elevated temperatures. This thermal decomposition of quaternary ammonium hydroxide to an olefin and an amine proceeds via an E2 mechanism and is referred to as the Hofmann elimination [31]. Upon preparation of the PEMEDA-β-CD diiodide, we experienced some partial decomposition of our material while drying the final product at 60 °C. We decided to look at the thermal stability in more detail. An analog with longer propylene linker (PEMPDA-β-CD diiodide), connecting the charged nitrogen atoms, was prepared for this reason. A higher stability of this derivative was presumed, because of the higher distance between the charged nitrogen atoms. The preliminary experimental setup consisted of heating the aqueous solution of both isomers to 80 °C with 1 equivalent of NaOH. The decomposition process was conveniently monitored by TLC, and revealed a higher stability of the PEMPDA-β-CD derivative. The TLC after 20 h showed a spot of the starting compound, while the degradation of PEMEDA-β-CD was already complete. We managed to separate the degradation products on a column of silica gel and identified them by ESIMS (Scheme 1). To determine the decomposition half-life of each isomer, we set up a ^1^H NMR experiment at elevated temperature (50 °C). Spectra of the sample solutions in D_2_O, with 20 equivalents of NaOH were acquired every hour in a course of 36 hours. The decreasing values of the integral intensity of CH_3_ protons of the substituent were plotted against time and kinetic curves for each derivative were obtained (Figure 1). Data from the plot clearly indicate that PEMPDA-β-CD is the more stable derivative. Values of the decomposition time constants were calculated by fitting the experimental data by a monoexponential function and are 7.9 h and 20.1 h for the PEMEDA-β-CD and PEMPDA-β-CD, respectively. Most probably, the closer proximity of the two cationic nitrogen sites, in the ethylene-linked substituent of the PEMEDA-β-CD diiodide, causes the stronger electrostatic repulsion which leads to a more rapid formation of the corresponding olefin. Based on these results the PEMPDA-β-CD was selected for further measurements.

### Inclusion properties of PEMPDA-β-CD in solution

**Scheme 2:**
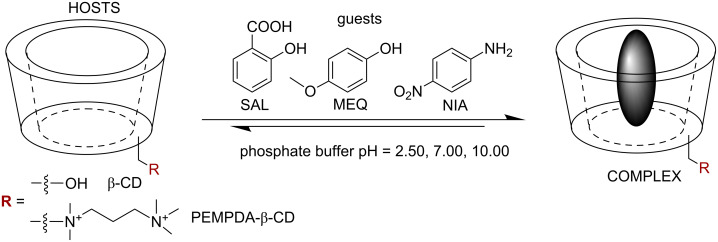
Host and guest molecules employed in *K*_s_ determination in solution at different pH.

To evaluate the ability of the cationic derivative PEMPDA-β-CD to form inclusion complexes in aqueous solution, we measured the stability constants (*K*_s_) with three model aromatic guest molecules (salicylic acid – SAL, *p*-methoxyphenol – MEQ, *p*-nitroaniline – NIA) at three different pH values (2.50, 7.00, 10.00) (Scheme 2).

The *K*_s_ values were obtained by isothermal titration microcalorimetry (ITC) [32–33], which was selected as the most suitable method for our purpose. Other methods were also tested, but their usability was found to be lower than ITC. For example ^1^H NMR spectroscopy [34] requires relatively high concentrations of the guest and host molecules, which are often impossible to achieve due to the poor solubility in aqueous media. Conventional UV–vis spectroscopy [35] was found difficult to be applied as well, because we were not able to achieve zero absorbance of the host solution (aqueous PEMPDA-β-CD dichloride), which is required to carry out the titration of guest solution.

**Figure 2:**
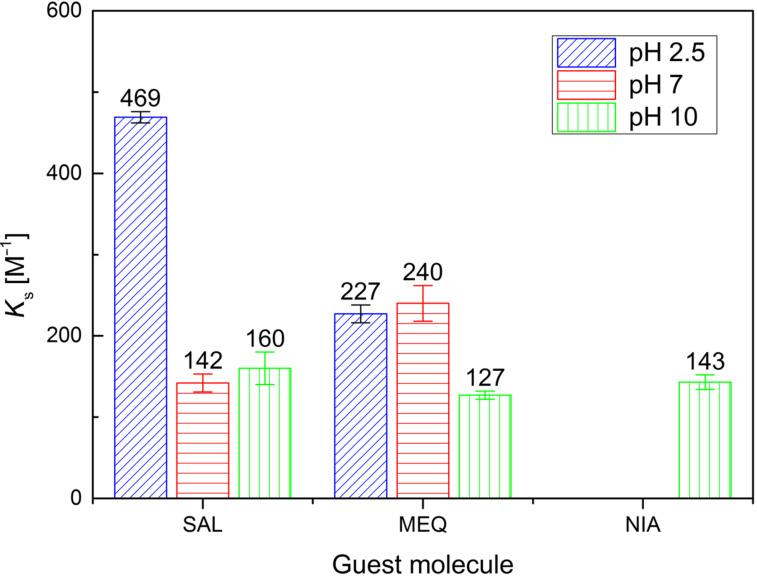
Stability constants for β-CD with SAL, MEQ and NIA obtained by ITC measurements.

**Figure 3:**
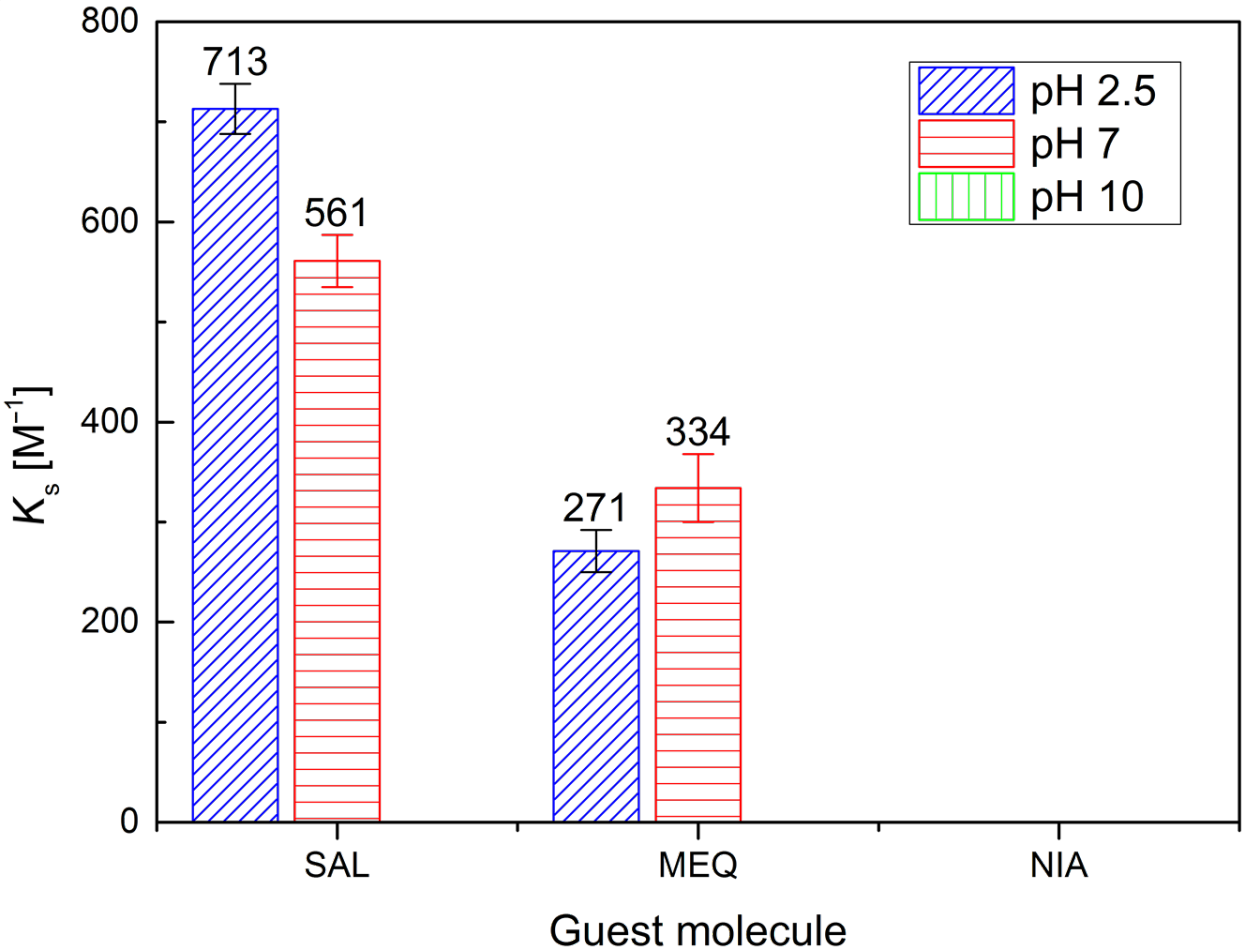
Stability constants for PEMPDA-β-CD with SAL, MEQ and NIA obtained by ITC measurements.

The *K*_s_ values for β-CD and PEMPDA-β-CD diiodide from ITC measurements are summarized in Figure 2 and Figure 3 respectively. The stoichiometry of all of the employed complexes was found to be 1:1. The *K*_s_ calculated for β-CD are in agreement with the literature [36–39]. The most stable complex of β-CD was obtained with SAL at pH 2.50 (*K*_s_ = 469 ± 7 M^−1^), whilst no association was detected with SAL and MEQ at pH 10.00. NIA was complexed by β-CD only at pH 10.00 (Figure 2). The collected data comply with the concept, that uncharged neutral molecules fit the best into the CD’s lipophilic inner cavity.

In the case of positively charged PEMPDA-β-CD, again the highest *K_s_* value was received for SAL at pH 2.50 (Figure 3). This may be explained by the contribution of the ion–dipole interaction between the positively charged substituent of the host and the carboxylic group of SAL, similarly as described in the literature [40]. Also the values of *K*_s_ of the PEMPDA-β-CD with MEQ in acidic and neutral solutions were higher than those obtained for the β-CD. Complexation of NIA with PEMPDA-β-CD was not observed as well as binding of SAL and MEQ at basic pH. In conclusion, we can state that the monosubstituted derivative PEMPDA-β-CD is able to form inclusion complexes with SAL and MEQ at acidic and neutral pH, whose stabilities are superior to those of the native β-CD.

### Immobilization of PEMPDA-β-CD on anionic surface via ionic self-assembly

**Scheme 3:**
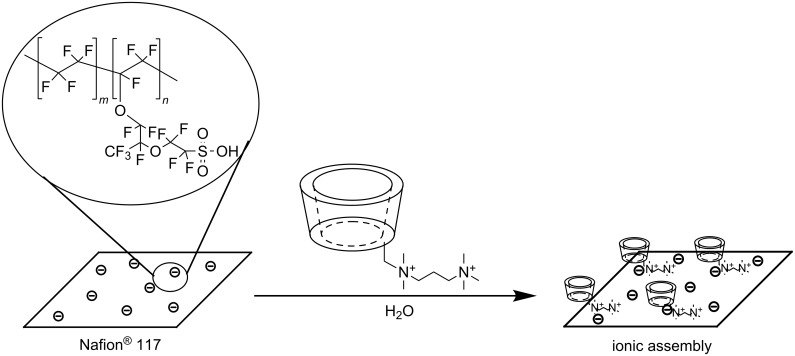
Deposition of PEMPDA-β-CD onto solid surface (Nafion^®^ 117).

In the next part of our research, we focused on the possibility of deposition of the permanently charged PEMPDA-β-CD onto the solid surface of a model anionic polymeric Nafion^®^ 117 membrane via simple ionic interactions (Scheme 3). Nafion^®^ is a sulfonated tetrafluoroethylene fluoropolymer-copolymer, especially favorable for our purpose because of its defined structure, with a known number of –SO_3_H groups (equivalent weight – EW = 1100 g·mol^−1^) and the absence of aromatic groups, which could be encapsulated by the CD cavity and negatively influence the complexation properties of the deposited CD. Preliminary experiments were performed on a strong cation exchange resin (Dowex^®^ 50), which has the chemical constitution of sulfonated polystyrene. It showed high deposition of cationic PEMPDA-β-CD, but also of the native β-CD. The results indicated that undesired inclusion of the styrene moieties in the CD cavity takes place, which blocks the cavity from further complexation of guest molecules from solution. At this point we replaced Dowex^®^ 50 for aliphatic Nafion^®^ 117. A cut-out of the foil (100 mm^2^, 35 mg) in H^+^ cycle was stirred in aqueous solution of the PEMPDA-β-CD and provisional data were collected by monitoring the decrease of the concentration by TLC and gravimetry. TLC indicated completion of the immobilization after 48 h and the amount of deposited PEMPDA-β-CD diiodide determined by gravimetry was 5.0 mg.

**Figure 4:**
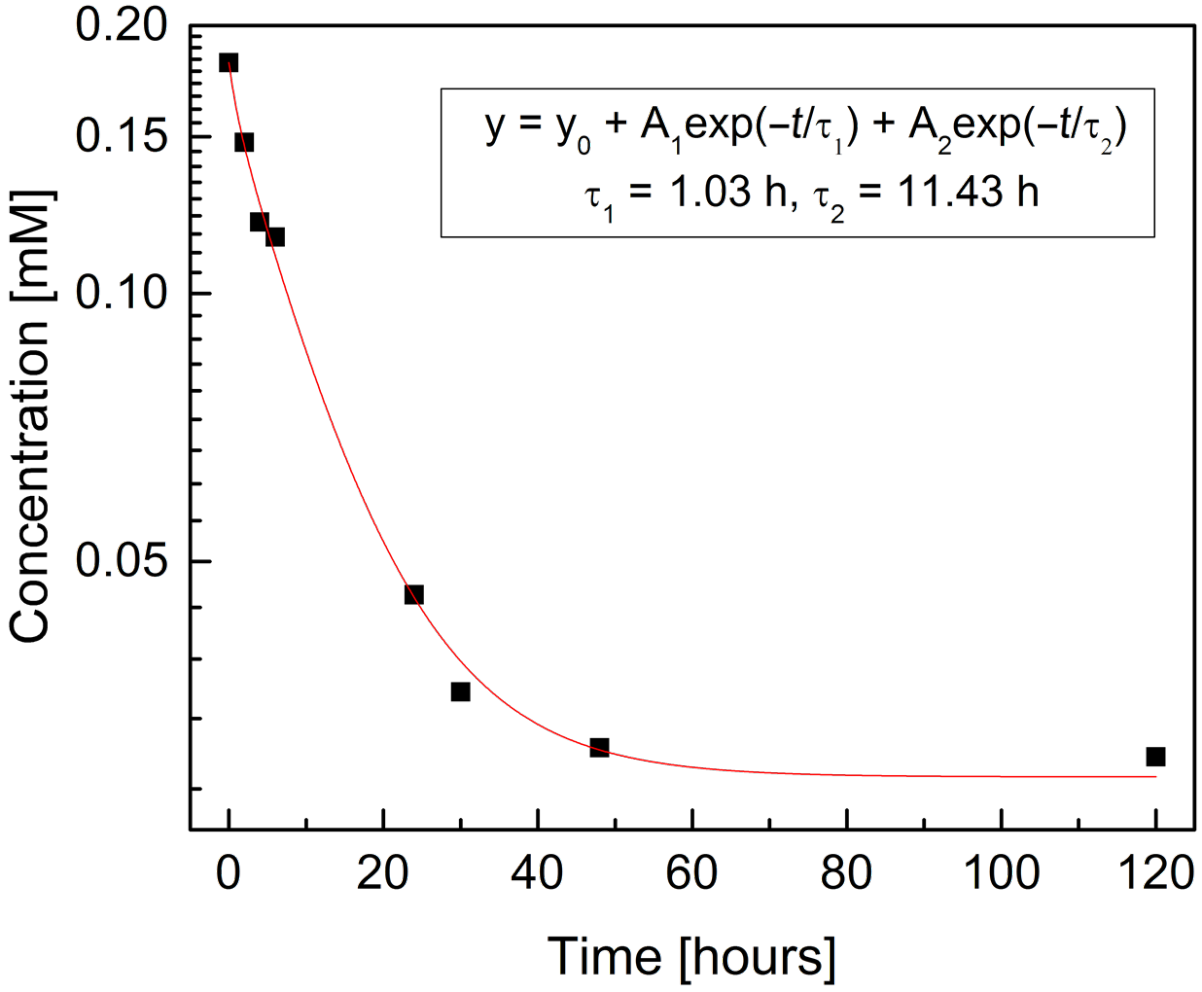
Deposition kinetics of PEMPDA-β-CD onto Nafion**^®^** 117 as obtained from ELSD detection of the decreasing concentration in the solution.

The amount of the immobilized PEMPDA-β-CD along with the deposition kinetics was determined by monitoring of the concentration decay by an evaporative light scattering detector (ELSD). The initial concentration of PEMPDA-β-CD was 0.2 mM (5.0 mg dissolved in 16.5 mL H_2_O). Nafion^®^ 117 H^+^ foil (100 mm^2^) was added to the solution and the deposition rate was monitored by direct injection of the reaction mixture in the ELSD input. Measured peak areas (area under curve – AUC) were converted to the concentrations and plotted against time to receive the deposition kinetics (Figure 4). From the log *c* vs *t* plot it follows, that the deposition kinetics is governed by a two-exponential process. The deposition time constants were calculated by fitting the experimental data by two exponential decay functions. Two time constants – τ_1_ = 1.03 h and τ_2_ = 11.43 h were obtained. The final residual equilibrium concentration was 0.03 mM, which was attributed to the signal of the nascent HI which is formed during the immobilization and its signal is picked up by the ELSD detector. Overall 10 mol % of available –SO_3_H groups were saturated by cationic CD derivative. These results were confirmed by repeated experiments and composition of the assembly was reproducible, also when using Nafion^®^ 117 in the NH_4_^+^ cycle. The attempts to achieve a higher degree of saturation of –SO_3_H groups by modifying the deposition conditions – saturated solution of PEMPDA-β-CD, higher temperature, longer deposition times – were not successful.

The assembly exhibits high overall stability. Attempts for desorption of the absorbed PEMPDA-β-CD were performed. The experiment consisted of stirring the Nafion^®^ cut-outs with immobilized PEMPDA-β-CD (5 mg) overnight in different solutions which were selected as capable of disrupting the ionic interactions between the support and CD (water, 5% and 10% aqueous NH_4_OH, 5% and 10% aqueous NH_4_HCO_3_). The possible desorption of the deposited CD was monitored by TLC. No leaching of the deposited CD could be observed, except in the case of 10% aqueous NH_4_HCO_3_, where some insignificant traces of PEMPDA-β-CD were detected by TLC.

### Inclusion of model guest molecules from the solution in the cavities of Nafion^®^-bound PEMPDA-β-CD

**Scheme 4:**
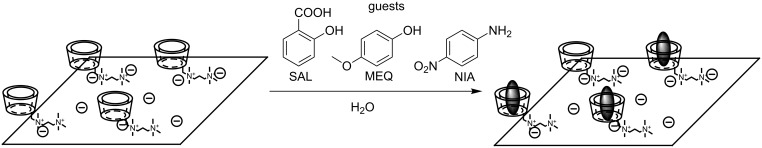
Deposition of three model guests into the cavities of immobilized PEMPDA-β-CD.

The ability of the prepared ionic assembly, composed of the cationic monosubstituted β-CD derivative anchored to the anionic surface, to encapsulate suitable guest molecules from the solution was finally studied. Three model guest molecules (SAL, MEQ, NIA) were employed in the experiment, so the acquired data can be compared with the results of inclusion obtained in the solution. The cut-outs of Nafion^®^ 117 in NH_4_^+^ cycle (100 mm^2^, 35 mg) with deposited PEMPDA-β-CD (5 mg) were stirred in the solution of the guest for 20 h, to reach equilibrium inclusion (Scheme 4). Subsequently the assembly was washed with H_2_O (5 × 3 mL) to remove the unspecifically bound guest and then the included guest was extracted from cavities by MeOH (1 × 3 mL). The amount of complexed guest was quantified by UV–vis spectroscopy of the MeOH extracts against the blank sample which consisted of Nafion^®^ 117 with no deposited PEMPDA-β-CD. This method proved to be very useful and simple tool for determination of the amount of included guests in the cavities of CD anchored to the surface.

For the sake of clarity we defined a new unit of measure, which describes the extent of inclusion of the guest in Nafion^®^-bound PEMPDA-β-CD. *ROC* (ratio of occupied cavities) is defined as a molar percentage of the cavities forming inclusion complex (Equation 1). In other words, the final number describes the percentage of the cavities saturated by the guest molecule.

[1]
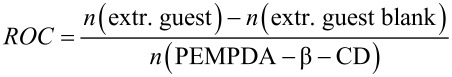


Here *n*(extr. guest) is the number of moles of guest extracted by MeOH from the Nafion^®^-bound PEMPDA-β-CD, *n*(extr. guest blank) is the number of moles of guest extracted by MeOH from the unmodified Nafion^®^ and *n*(PEMPDA-β-CD) is the number of moles of deposited PEMPDA-β-CD.

The results are summarized in Table 1. The highest *ROC* was obtained for SAL (34.5%) which corresponds very well with the data from the measurement of complexation in the solution. *ROC* of MEQ (17.5%) is proportionally lower and also correlates nicely with the data from solution. Maximal solubility of NIA is lower than the ones of SAL and MEQ, for this reason we had to use a lower concentration of the incubation solution. When using the concentration of 3.20 mM we received a value of 10.3%. It is apparent, that *ROC* depends strongly on the initial concentration of the incubation solution. Binding in the solution with one-tenth of the maximum concentration of SAL or MEQ results in a *ROC* about ten times lower. In the case of NIA the dependence of the inclusion on guest concentration is even higher.

**Table 1:** Results of the inclusion of different guests on the Nafion^®^ 117 and PEMPDA-β-CD ionic assembly at neutral pH.

Guest	*c* (guest) M	*n* (included guest) mol	*n* (CD on surface)	*ROC* (%)

SAL	1.40 × 10^−2^	1.14 × 10^−6^	3.30 × 10^−6^	34.5
SAL	1.40 × 10^−3^	1.05 × 10^−7^	3.30 × 10^−6^	3.2
MEQ	1.40 × 10^−2^	5.76 × 10^−7^	3.30 × 10^−6^	17.5
MEQ	1.40 × 10^−3^	4.55 × 10^−8^	3.30 × 10^−6^	1.4
NIA	3.20 × 10^−3^	3.40 × 10^−7^	3.30 × 10^−6^	10.3
NIA	3.20 × 10^−4^	1.40 × 10^−8^	3.30 × 10^−6^	0.4

## Conclusion

In summary, we performed an NMR study about the thermal stability of two monosubstituted bis(tetraalkylammonium) CD derivatives PEMEDA- and PEMPDA-β-CD and selected the more stable analogue (with propylene linker) for further measurements. The next study was focused on the inclusion properties of PEMPDA-β-CD in solution, compared to the native β-CD, with three model guests. We found out, that the cationic derivative retains the inclusion properties and forms even more stable guest–host complexes than β-CD with SAL and MEQ at acidic or neutral pH. We developed a method for immobilization of PEMPDA-β-CD onto the solid surface of Nafion^®^ 117, along with determination of the surface coverage and deposition kinetics by ELSD. Saturation of 10 mol % of the available –SO_3_H with PEMPDA-β-CD was achieved repeatedly. Finally, the inclusion of three model guests on the assembly of Nafion^®^ 117 modified with PEMPDA-β-CD was conducted. A simple method of quantification of the extent of inclusion was developed using UV–vis spectroscopic measurements of the MeOH washes. The best results were obtained for SAL, where 34.5% of the available cavities of the PEMPDA-β-CD were in the inclusion complex form. The presented results are promising for the possible application of supramolecular ionic assemblies on solid surfaces as systems for prolonged and controlled release of active compounds or solid–liquid extraction systems.

## Supporting Information

File 1 Experimental part, NMR, and ITC data. General experimental procedures. Detailed experimental procedures for all of the measurements. Copies of ^1^H NMR spectra and ITC data.
