# Supplementary material for: Properties of cationic monosubstituted tetraalkylammonium cyclodextrin derivatives – their stability, complexation ability in solution or when deposited on solid anionic surface
Source: Beilstein J Org Chem. 2015 Feb 2;11:192–9. doi: 10.3762/bjoc.11.20 (PMC4362307; doi:10.3762/bjoc.11.20)
Supplement: File 1 — Experimental part, NMR, and ITC data. General experimental procedures. Detailed experimental procedures for all of the measurements. Copies of 1H NMR spectra and ITC data. [file Beilstein_J_Org_Chem-11-192-s001.pdf]

Supporting Information

for

Properties of cationic monosubstituted tetraalkylammonium cyclodextrin derivatives – their stability, complexation ability in solution or when deposited on solid anionic surface

Martin Popr[1], Sergey K. Filippov[2], Nikolai Matushkin[2], Juraj Dian[3] and Jindřich Jindřich[*,1]

Address: [1]Department of Organic Chemistry, Faculty of Science, Charles University in Prague, Hlavova 8, 128 40, Prague 2, Czech Republic, [2]Institute of Macromolecular Chemistry AS CR, v. v. i., Heyrovskeho nam. 22, 16206, Prague 6, Czech Republic and [3]Department of Chemical Physics and Optics, Faculty of Mathematics and Physics, Charles University in Prague, Ke Karlovu 3, 121 16, Prague 2, Czech Republic
Email: Jindřich Jindřich[*] - jindrich@natur.cuni.cz

[*]Corresponding author

Experimental part, NMR, and ITC data. General experimental procedures. Detailed experimental procedures for all of the measurements. Copies of ^{1}H NMR spectra and ITC data.

Content:

Instruments, general procedures and materials S1
Experimental part S2
References S6

Instruments, general procedures and materials

^{1}H NMR spectra were acquired on Bruker AVANCE III at 600 MHz. Mass spectra were obtained on Bruker ESQUIRE 3000 ES-ion trap instrument with electrospray ionization (ESI) in positive mode. All samples were dissolved in methanol. ITC measurements were carried out on GE MicroCal^{TM} iTC$_{200}$. ELSD measurement was performed using Shimadzu LC-20AD

HPLC pump and evaporative light scattering detector Alltech® 3300 ELSD. UV–vis spectra were acquired on THERMO Spectronic Heλios Gamma.

Thin layer chromatography (TLC) was performed on silica gel coated aluminium sheets DC-Alufolien Keisegel 60 F_{265} (Merck, Darmstadt, Germany). Dipping in 50% H_2SO_4 with subsequent carbonization by a heat gun was used for spot detection of all CD derivatives.

β-CD was purchased from WAKO Chemicals (Germany) and recrystallized from water prior use. Salicylic acid (SAL), *p*-methoxyphenol (MEQ), *p*-nitroaniline (NIA) were purchased from Sigma-Aldrich. Their identity and purity was checked by ^{1}H NMR and were used without further purification. PEMEDA- (6^I-(*N,N,N',N',N'*-pentamethylethane-1,2-diammonio)-6^I-deoxy-β-cyclodextrin) and PEMPDA-β-CD (6^I-(*N,N,N',N',N'*-pentamethylpropane-1,3-diammonio)-6^I-deoxy-β-cyclodextrin) diiodides were prepared by the procedure published recently by our group[1]. Nafion ® 117 membrane was obtained from Ion Power, Munich, Germany. Other chemicals were purchased from common commercial sources and used without further purification unless otherwise noted comply to the concept

Experimental part

Thermal stability of PEMEDA- and PEMPDA-β-CD

Experiment setup: 20.0 mg of each derivative was dissolved in 0.7 ml of D_2O and transferred to NMR tube. After performing lock and gradient shim, 20 equivalents of NaOH were added (0.2 ml of 7.5% NaOH in D_2O). First spectrum was acquired immediately after reaching 50 °C in the probe (individually) and then time-lapse experiment was started. Spectra were acquired after every hour in the course of 36 hours. Each spectrum was individually integrated. The decaying integral intensity of the CH_3 protons (around 3.6 ppm) was related to the integral intensity of H-1 protons (around 5.3 ppm), which remains constant and plotted against time.

Copies of the^{1}H NMR spectra:

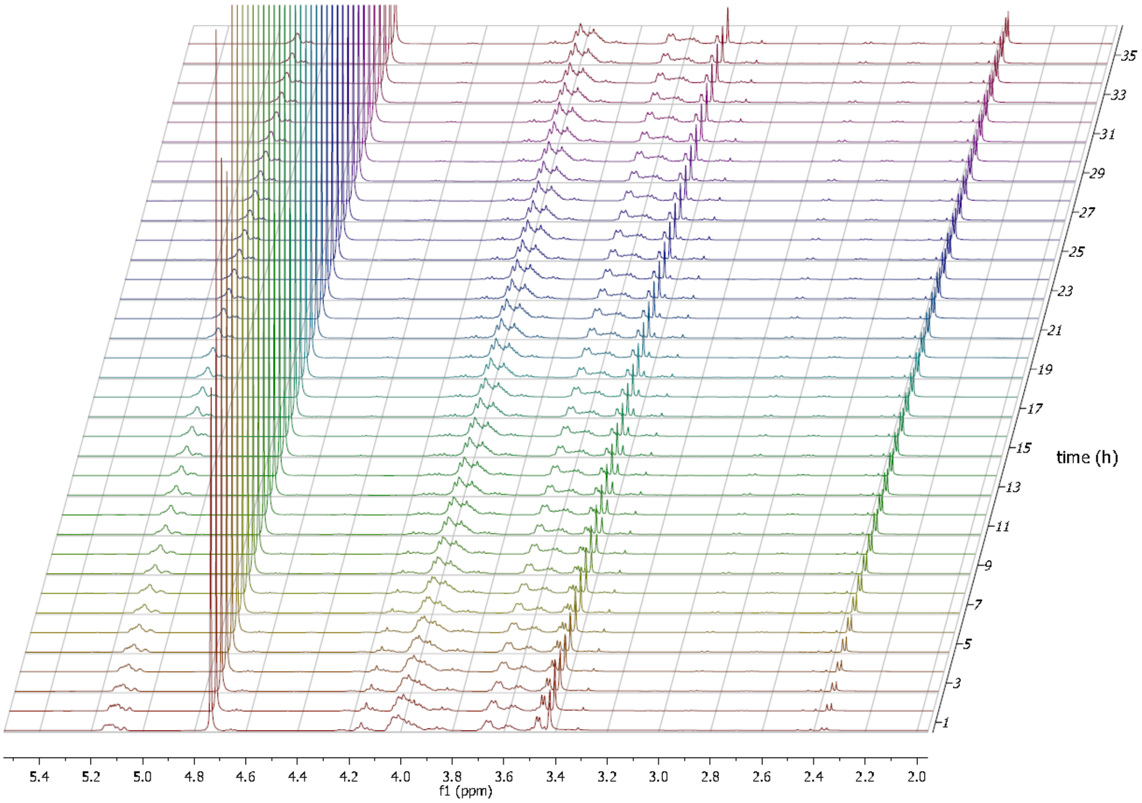

Spectra 1. ^{1}H NMR spectra illustrating the thermal decomposition of PEMEDA-β-CD.

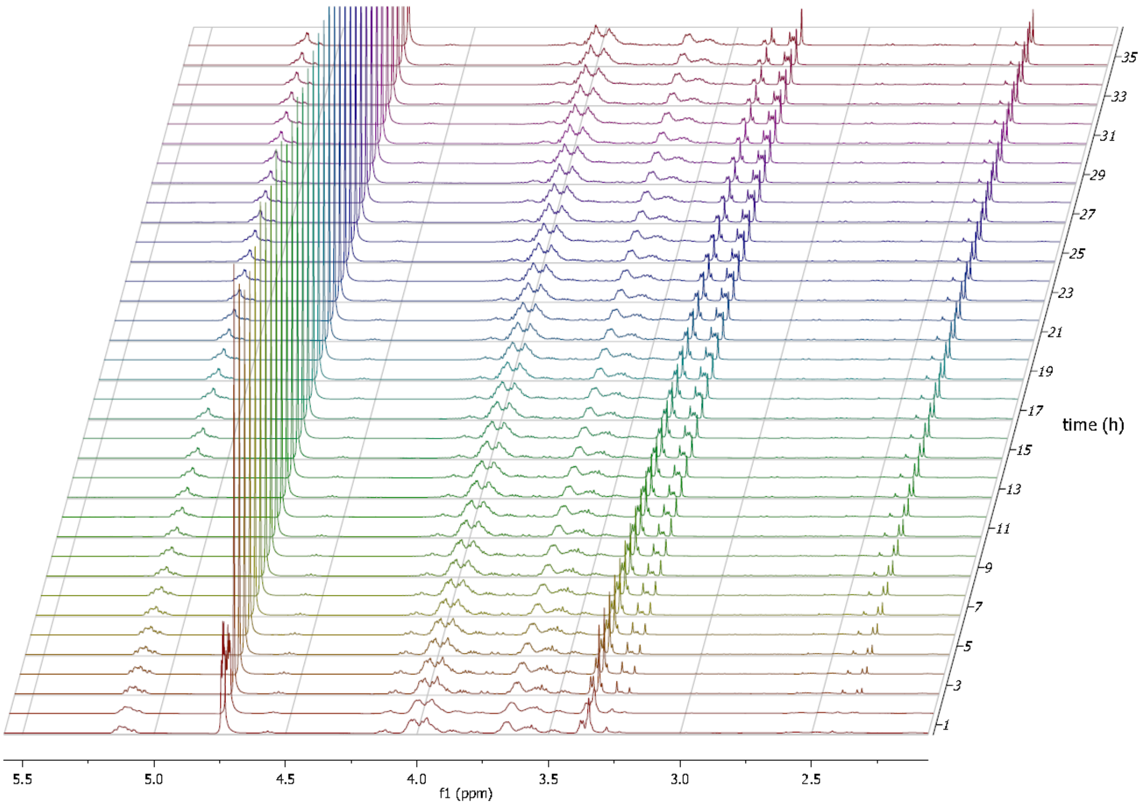

Spectra 2. ^{1}H NMR spectra illustrating the thermal decomposition of PEMPDA-β-CD.

Inclusion properties of PEMPDA-β-CD in solution - ITC

Experiment setup: Both guests and hosts were dissolved in phosphate buffer (c=0.02 M) of pH 2.50, 7.00 and 10.00. Host concentration was 8.81 mM (for β-CD) and 7.50 mM (for PEMPDA-β-CD diiodide). Guest concentrations were set always ten times lower (0.88 mM and 0.75 mM respectively). The experiment consisted of consecutive injections of the host solution (2 µl) into the calorimeter cell containing the guest solution (280 µl). Temperature of the cell was set to 25 °C. The time between injections was usually 5 min. The data were analyzed using Microcal ORIGIN software. The experimental enthalpy was obtained by integrating the raw data signal. The integrated molar enthalpy change per injection was obtained by dividing the experimentally measured enthalpy by the number of moles of substance added. The final enthalpograms are the plots of the integrated molar enthalpy as a function of total substance concentration in the calorimeter sample cell.

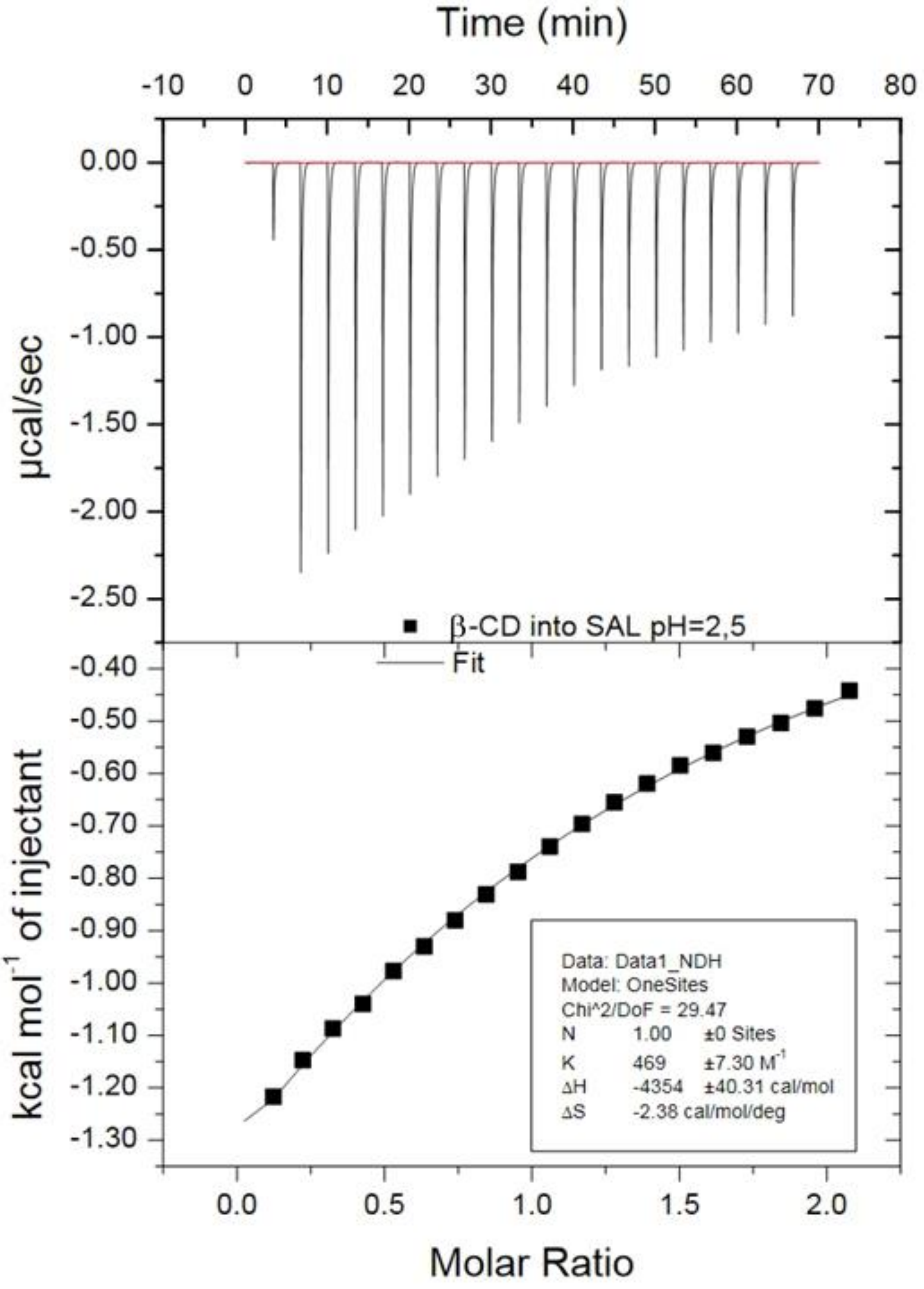

Figure 1. An example of the enthalpogram obtained from the ITC experiment for the titration of β-CD into SAL pH=2.50.

Immobilization of PEMPDA-β-CD on anionic surface via ionic self-assembly

Experimental conditions of the ELSD measurement: Only one compound was present in the solution (PEMPDA-β-CD), so the analysis was carried out without chromatographic column. Mobile phase was isocratic mixture of H_2O-MeOH 4:1, flow rate 1 ml/min, pressure about 15

bar. Injected volume was 20 µl. ELDS detector was set to the temperature of 39 °C, gas flow 1.3 l/h, gain 4. Calibration of PEMPDA-β-CD was performed using set of standard samples with known concentration, prior to the kinetics measurement.

Obtained values of decaying AUC (area under curve) were converted to concentrations using the calibration and plotted against time to receive the kinetics of the deposition on solid surface. The deposition time constants were calculated by fitting the experimental data by two exponential decay functions using Origin® software.

Inclusion of model guest molecules from the solution in the cavities of Nafion®-bound PEMPDA-β-CD

Experiment setup: Nafion cut-outs with immobilized PEMPDA-β-CD (5 mg) were neutralized by stirring in 3% NH_4OH (2 ml), prior to the measurement. UV–vis spectra of the H_2O and MeOH washes were acquired in the spectral range 200-400 nm. First, calibration of SAL, MEQ and NIA in H_2O and MeOH were carried out, along with the determination of linearity. Absorbance values were read in the absorption maxima near 300 nm.

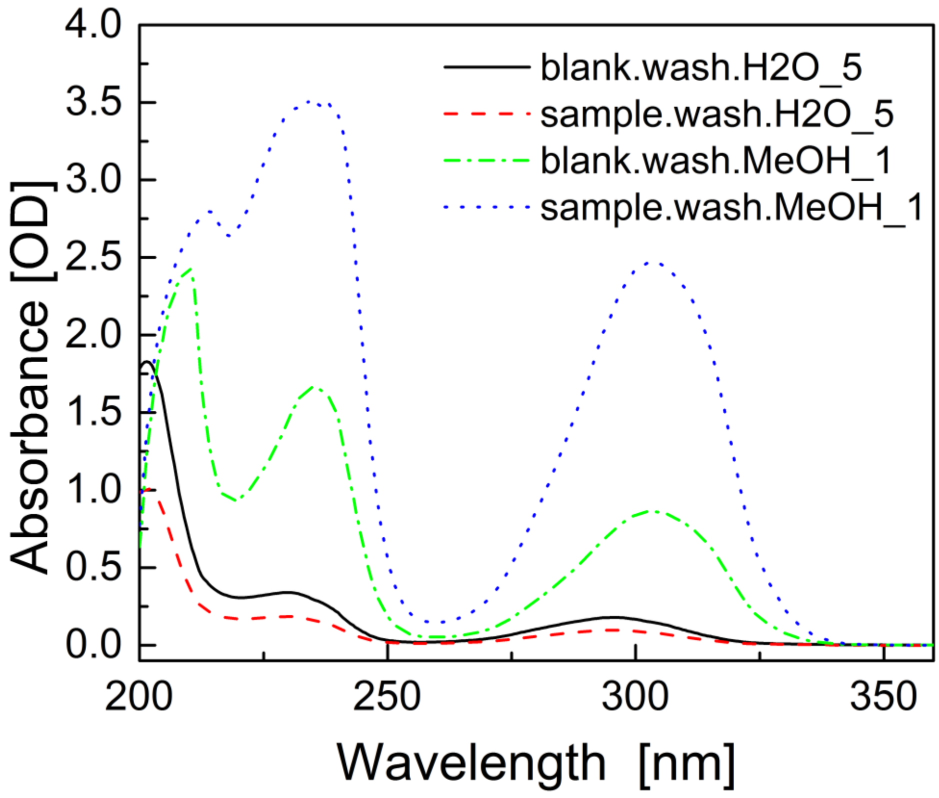

Figure 2. Example of UV–vis spectra of the H_2O and MeOH washes for inclusion of SAL

References

1. Popr, M.; Hybelbauerová, S.; Jindřich, J. *Beilstein J. Org. Chem.* **2014**, *10*, 1390–1396.
